# Spin filtering with Mn-doped Ge-core/Si-shell nanowires†

**DOI:** 10.1039/c9na00803a

**Published:** 2020-02-28

**Authors:** Sandip Aryal, Ranjit Pati

**Affiliations:** Department of Physics, Michigan Technological University Houghton MI 49931 USA patir@mtu.edu

## Abstract

Incorporating spin functionality into a semiconductor core–shell nanowire that offers immunity from the substrate effect is a highly desirable step for its application in next generation spintronics. Here, using first-principles density functional theory that does not make any assumptions of the electronic structure, we predict that a very small amount of Mn dopants in the core region of the wire can transform the Ge–Si core–shell semiconductor nanowire into a half-metallic ferromagnet that is stable at room temperature. The energy band structures reveal a semiconducting behavior for one spin direction while the metallic behavior for the other, indicating 100% spin polarization at the Fermi energy. No measurable shifts in energy levels in the vicinity of Fermi energy are found due to spin–orbit coupling, which suggests that the spin coherence length can be much higher in this material. To further assess the use of this material in a practical device setting, we have used a quantum transport approach to calculate the spin-filtering efficiency for a channel made out of a finite nanowire segment. Our calculations yield an efficiency more than 90%, which further confirms the excellent spin-selective properties of our newly tailored Mn-doped Ge-core/Si-shell nanowires.

## Abstract 2

Incorporating spin functionality into a semiconductor core–shell nanowire that offers immunity from the substrate effect is a highly desirable step for its application in next generation spintronics.

## Graphical abstract

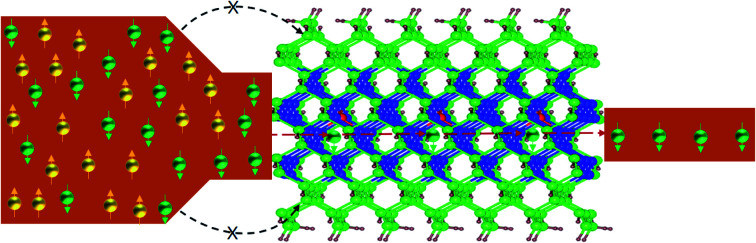

## Introduction

Since their inception,^1^ core–shell semiconductor nanowires, built of group IV elements such as Ge and Si, are the subject of immense interest.^2–18^ This level of interest in these nanostructures can be attributed to their multi-functional applications ranging from next-generation electronics,^3,12,19^ to biosensors^20^ to photovoltaics^21,22^ to quantum computing devices.^4,11,17^ For example, Ge-core/Si-shell nanowires, which are the materials of choice due to their compatibility with the current Si-based technology, have been successfully synthesized in high yield^1–3^ and reported to exhibit ballistic transport at a low bias with a scattering mean free path of ∼500 nm.^2,3^ Converting these low dimensional semiconductors to spin active structures would offer an additional opportunity for using them in spin-based electronics of the future.^23–27^ Intentional addition of a small number of magnetic impurities^28,29^ would be a viable path to implement spin functionality into such a system without destroying completely its semiconducting properties. In fact, there have been numerous studies of Mn dopants in Si and Ge nanowires,^30–37^ nano-columns^38^ and nanotubes.^39^ Depending upon the concentration of Mn, they have been reported to exhibit ferromagnetism at room temperature.

However, unlike these homogeneous nanowires, where the stabilization of the ferromagnetic phase at room temperature is a major challenge due to the substrate effect and often requires alloying, doping Mn into the core region of a Ge–Si core–shell heterostructure nanowire would offer significant advantages. Due to the valence band offset between the Ge and Si in core–shell nanowires, spin carriers in the Mn-doped core–shell structure can be guided through the spin active Ge core of the wires resulting in complete suppression of spin lifetime degradation due to scattering and recombination with the surface states. Furthermore, due to the confinement of carriers to the core region, we could limit the momentum dependent randomization of spins (spin dephasing) during spin transport – an important prerequisite in spintronics.^24^ Mn-doped core–shell channels can also alleviate the conductivity mismatch challenge associated with the Schottky junction at the semiconductor nanowire/metal interface. Despite these advantages, until now, no efforts are made in understanding the role of Mn dopants on carrier transport at a Ge-core/Si-shell nanowire junction.

In this work, we have used predictive first-principles density functional theory (DFT) to investigate the electronic structure and magnetic properties of Mn-doped Ge-core/Si-shell nanowire hetero-structures. We limit ourselves to a low concentration of Mn dopants in the Ge core part of the core–shell structure due to its low solubility in semiconductors.^40^ Our calculations reveal that the addition of Mn dopants transforms the semiconducting Ge–Si core–shell nanowire into a stable half-metallic ferromagnet. The energy band diagram yields a semiconducting behavior for one spin direction while the metallic behavior for the other. Inclusion of spin–orbit (SO) interaction is found to have a minimal effect on the energy band structure; a maximum SO splitting of ∼24 meV is obtained at the crossing points of majority and minority bands. Subsequently, a quantum transport approach^41^ is used to calculate spin-polarized transmission in a prototypical Mn-doped Ge-core/Si-shell nanowire junction to assess its usage in a spin-filtering device. A spin filter efficiency of 90.4% is found, further confirming the spin selective properties of this material.

## Computational details

We have considered a Ge-core/Si-shell nanowire along the 〈110〉 direction as it has been reported to be the preferred growth direction for a diameter of less than 20 nm.^3^ Since Mn prefers the substitutional site in Ge,^33,42–44^ we replace one of the Ge in the core region of the unit cell with a Mn atom. In order to avoid the undesirable scattering of the carriers during transport, the surface dangling bonds in the Mn-doped Ge-core/Si-shell nanowire are passivated by hydrogen atoms. A supercell is constructed by placing a unit cell comprised of 47 Ge, 80 Si, 48 H, and 1 Mn in a rectangular grid with the nanowire wall to wall distance (along *x* and *y* axes) greater than 11 Å between the cells (ensuring negligible interaction between the nanowire and its replica). The infinite nanowire is built by stacking up the supercell in the *z*-direction; the percentage of Mn atoms in the 128-atom (excluding hydrogen atoms) unit cell is 0.78%. Subsequently, the nanowire geometry is optimized and the electronic structure and magnetic properties are calculated using the plane-wave basis function and the spin-polarized density functional method as implemented in the Vienna *Ab initio* Simulation Package (VASP).^45,46^ The generalized gradient approximation (GGA) in the form of the Perdew–Burke–Ernzerhof (PBE) functional^47^ is used to approximate the exchange–correlation potential. To correct the self-interaction error associated with the use of the PBE functional, we have also used a hybrid functional (HSE06) (ref. 48 and 49) that blends part of the Hartree–Fock exchange with the exchange and correlation potential from the PBE functional. The projector augmented wave (PAW) pseudopotential is used to model the valence–core interactions. During the geometry optimization, structural relaxations that include the strain effect due to the Mn dopants and lattice mismatch between Si and Ge are carried out without symmetry constraint until the residual force on each atom reduces to 0.01 eV Å^−1^; the convergence criterion for total energy is set at 10^−7^ eV. The Monkhorst–Pack (MP) *k*-point mesh of 1 × 1 × 7 and a kinetic energy cut-off value of 400 eV are used for these calculations. A non-collinear spin-polarized calculation that includes spin–orbit interaction is also performed to measure the spin–orbit coupling induced splitting of energy bands.

To examine the spin-filtering properties of this material in a device configuration, we have constructed a prototypical Mn-doped Ge-core/Si-shell nanowire junction; a finite segment of the nanowire is sandwiched between two metallic gold electrodes with an electrode–electrode distance of ∼2.37 nm. To avoid charge trapping at the lead-nanowire interface, we have passivated the unsaturated dangling states of the finite nanowire at the interface by H atoms as done for the surface states. A real space orbital dependent spin unrestricted DFT approach is used to construct the spin-polarized retarded Green's function (*G*^*σ*^) for the open junction^41^ by dividing it into two parts: (a) an active scattering part consisting of the finite nanowire channel and 38 atoms from the gold lead, and (b) an unperturbed gold lead that retains its bulk properties. The inclusion of gold atoms from the lead during the self-consistent calculation allows us to include explicitly the charging effects due to coupling with the semi-infinite electrodes. We have used a *posteriori* hybrid B3LYP^50^ exchange–correlation functional that partly corrects the self-interaction error for this calculation. This hybrid functional has been shown to give a much better description of transmission than the pure functional.^51^ In addition, a recent density of states analysis in transition metal compounds^52^ has shown that the B3LYP results agree well with the results obtained from embedded dynamical mean-field theory. The convergence thresholds for total energy, root mean square and maximum density are set at 10^−9^, 10^−8^, and 10^−6^ a. u. respectively. An all-electron 6-311g* Gaussian basis function^50^ is used for Mn and H. For practical purposes, the Ge, Si, and Au atoms are represented with a LANL2DZ effective core-potential basis set.^50^ Subsequently, the transmission function for the majority and minority spin carriers is calculated as a function of injection energy using a spin-conserved tunneling approach^41^ that does not take into account the incoherent spin-flip scattering effect. The details of our method can be found in our previous work.^41^

## Results and discussion

**Fig. 1:**
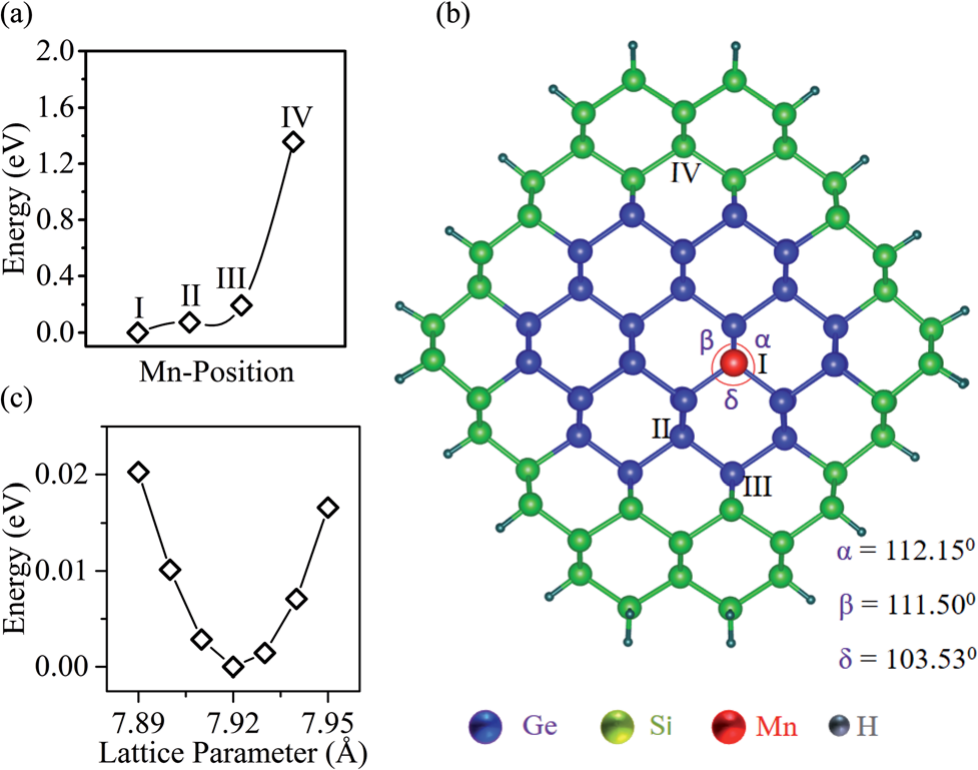
(a) Energy *vs.* Mn position in the nanowire; I, II, III, and IV refer to the Mn positions in the schematic (b). (b) The top view of the optimized Mn-doped Ge-core/Si-shell nanowire along the 〈110〉 direction. The core diameter of the nanowire is 11.7 Å; the unsaturated surface states are passivated by H-atoms. (c) Energy *vs.* lattice parameter of the nanowire. The minimum energy is set to zero in the energy scale for both (a) and (c).

We begin by examining the energy of the Mn-doped Ge-core/Si-shell nanowire when a Ge atom at various sites in the nanowire is replaced by a Mn atom. Our calculations reveal that the energy of the nanowire increases as we dope Mn away from the center of the core in the radial direction (Fig. 1a). The energy barrier for Mn in going from the core position I to the shell position IV is ∼1.4 eV, suggesting that the core positions are the preferred positions for Mn. The most energetically stable nanowire structure is illustrated in Fig. 1b. The total energy *vs.* the lattice parameter curve (Fig. 1c) is calculated to determine the equilibrium lattice parameter *a* of the nanowire. The *a* value is found to be 7.92 Å in the doped nanowire (Mn at site I), which is 0.01 Å shorter than that in the un-doped Ge–Si core–shell nanowire of a similar dimension. The bond distance between the Mn and the nearest neighbor Ge (2.42 Å) is found to be 0.04 Å shorter than the Ge–Ge bond distance (2.46 Å) of the un-doped nanowire indicating a lateral bond strain of ∼1.6% upon substitutional doping of Mn. This is expected because we replace the Ge with a larger electron cloud (*Z* = 32) by the smaller Mn (*Z* = 25). From bond angle analysis (Fig. 1b), we find that the tetrahedral symmetry around Ge is distorted upon the substitution of an Mn; the maximum angular deviation of 5.5% is found at the Mn site.

**Fig. 2:**
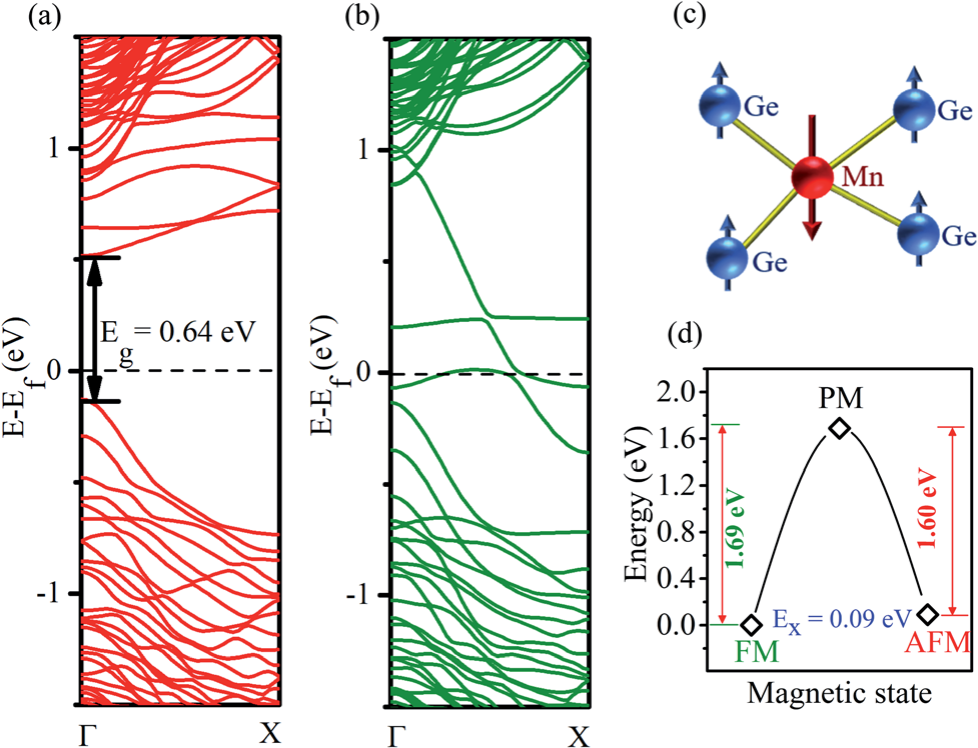
Electronic band structure (PBE) of the Mn-doped Ge-core/Si-shell nanowire: (a) minority-spin direction and (b) majority-spin direction. (c) Schematic showing the local alignment of magnetization of Mn and nearest Ge atoms in the nanowire. (d) Calculated energy for paramagnetic (PM), ferromagnetic (FM), and antiferromagnetic (AFM) configurations; the energy of the most stable FM state is set to zero.

Next, we comment on our calculated electronic structure (Mn at site I). Upon substitutional doping of Mn, the strong exchange interaction arising from the unpaired d-electrons of Mn splits the spin-degenerate energy bands of the Ge-core/Si-shell nanowire into the majority and minority spin bands (shown in Fig. 2). A half-metallic feature is clearly noticeable in Fig. 2. The minority spin electrons (Fig. 2a) exhibit a semiconducting behavior with an energy gap of 0.64 eV. The valence band maximum and the conduction band minimum are found at the same *Γ* point confirming the direct nature of the bandgap as found for the un-doped Ge-core/Si-shell nanowire. The un-doped nanowire of a similar diameter is reported to have a direct bandgap of 0.89 eV.^19,53^ The majority spin carriers (Fig. 2b), however, show a metallic behavior. Analysis of the atom decomposed band structure for a minority spin direction (see Fig. S2a in the ESI†) reveals that the contributions to the valence band at the *Γ* point mainly come from the Ge atoms. However, in the case of the conduction band (CB), Mn and Ge contributions are comparable at the high symmetry *Γ* point. As we move to the next higher energy band (CB + 1), the contribution of the Mn dominates over Ge at the *Γ* point. For the majority spin case (see Fig. S2b in the ESI†), both Ge and Mn contribute to energy bands near the Fermi level. The magnetization is found to be localized around the Mn atom. The contribution to the magnetization mainly comes from the d orbital of Mn atoms and is found to be −3.18 μ_B_ in the case of the PBE functional. Though relatively small in magnitude (∼0.08 μ_B_), the nearest neighbor Ge atoms are found to have magnetizations of opposite sign that comes from their p states. The local magnetic alignments in the vicinity of the Mn atom are shown in Fig. 2c. Similar local antiparallel magnetic alignments have been reported previously in Mn-doped systems.^29,33^ To understand the strength of exchange interaction between the Mn dopants, we double the unit cell size with the same Mn coverage and recalculated the energy (at zero temperature) for ferromagnetic (FM) and anti-ferromagnetic (AFM) coupling between the Mn atoms. The FM state is found to be lower in energy than the AFM state by 90.2 meV. A similar order for exchange energy is reported in Mn-doped homogeneous nanowires.^33^ For practical application at room temperature, however, we need to understand the thermodynamic stability of these magnetically ordered states. We calculated the metastable high entropy paramagnetic state (the expected transition point between FM and AFM states) to estimate the energy barrier (shown in Fig. 2d). The energy barrier is found to be 1.69 eV, which is much higher than that at room temperature (26 meV), suggesting that the FM ordering found here is stable at room temperature.

**Fig. 3:**
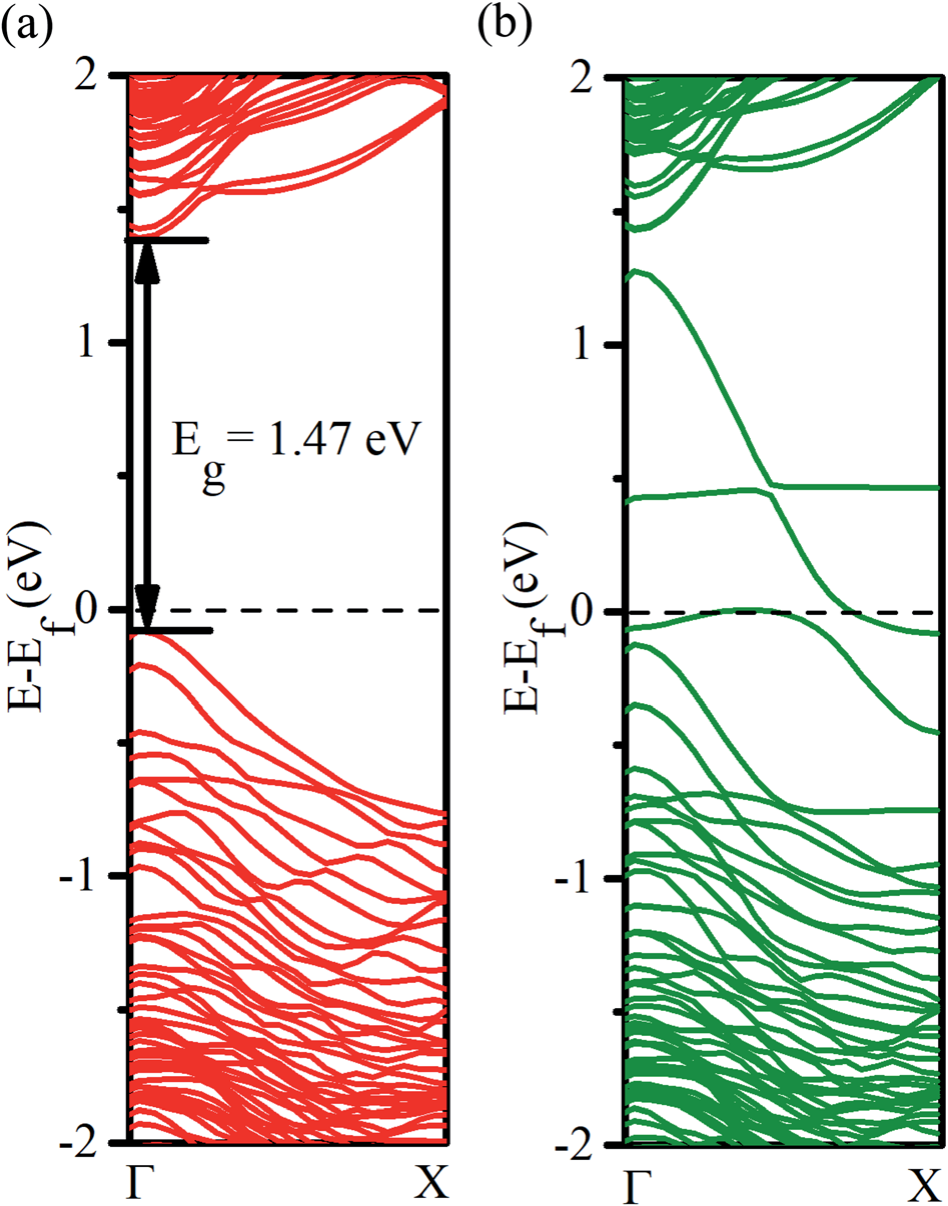
Electronic band structure (HSE06) of the Mn-doped Ge-core/Si-shell nanowire: (a) minority-spin direction and (b) majority-spin direction.

To gauge the effect of self-interaction error associated with the use of the PBE functional for the exchange–correlation energy and confirm the half-metallic properties of our system, we have also performed the band structure calculation using a hybrid functional (HSE06). It has been reported that the use of HSE06 yields bandgaps closer to experimental values in group IV semiconductors.^54^ Our results are presented in Fig. 3. For the minority spin direction, a comparison of the band diagrams obtained using the PBE functional (Fig. 2a) and HSE06 hybrid functional (Fig. 3a) reveals an increase in the bandgap from 0.64 eV to 1.47 eV upon correcting (partly) the self-interaction error. However, the observed metallic behavior for the spin majority case (Fig. 3b) is not affected by the self-interaction correction. The magnetic moment of the Mn is found to be −3.96 μ_B_, which is higher in magnitude than that found with the use of the PBE functional. The nearest neighbor Ge atoms are found to have oppositely aligned magnetic moments (∼0.13 μ_B_) as observed for the PBE functional.

**Fig. 4:**
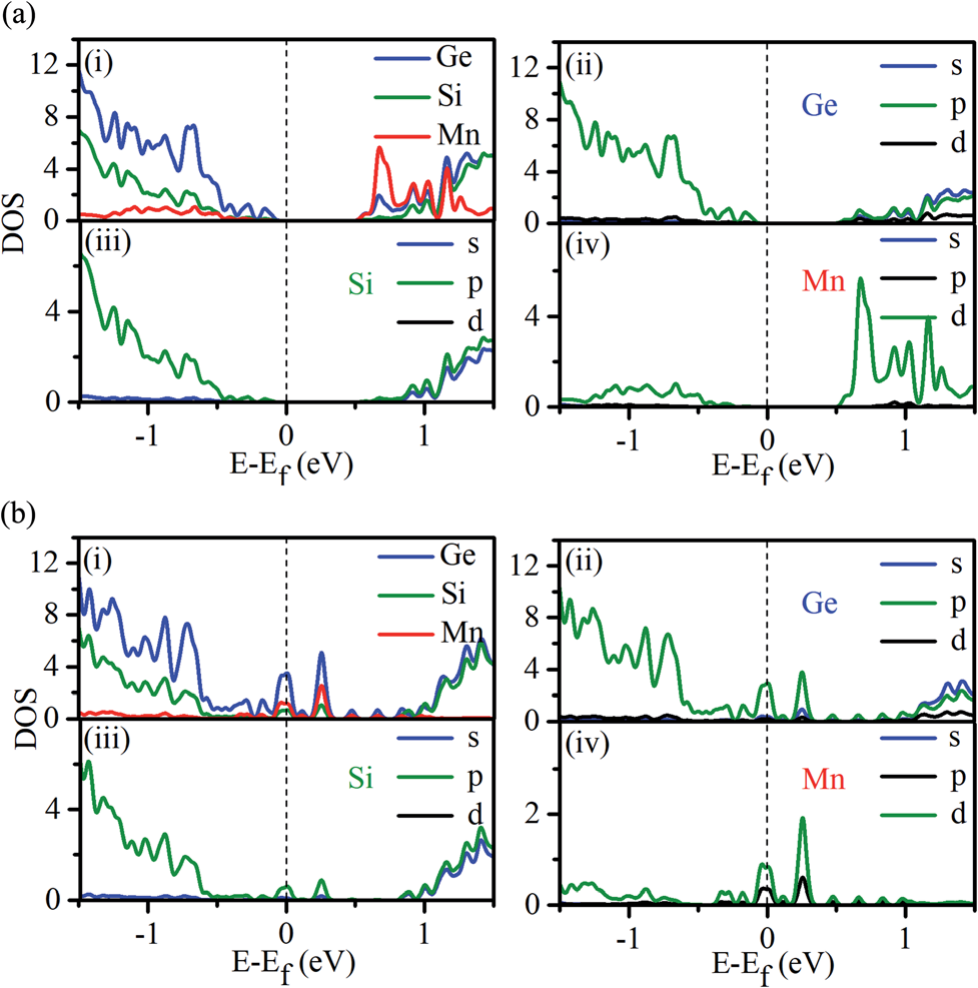
Atom and orbital decomposed density of states (DOS) of the Mn-doped Ge-core/Si-shell nanowire: (a) minority spin direction and (b) majority spin direction.

To gain a deeper insight into the origin of the observed half-metallic behavior, we have calculated the spin-polarized atom decomposed and orbital decomposed density of states (DOS) for Mn-doped nanowires. The results obtained using the PBE functional are presented in Fig. 4. In the minority spin case (Fig. 4a), the energy gap is noticeable, which further confirms its semiconducting behavior; the Fermi level lies in the gap. The valence band is clearly dominated by Ge, as observed from the atom decomposed band structure (see Fig. S2a and S3a in the ESI†). For the majority spin direction (Fig. 4b), a finite DOS at the Fermi energy confirms its metallic character. Orbital decomposed DOS reveals that the hybridization of the d state (with some p contribution) of Mn and the p state of Ge is responsible for the metallic character, which is also evident from the atom decomposed band structure (see Fig. S2 and S3 in the ESI†). The absence of energy states in the spin minority case and finite DOS in the spin majority case at the Fermi energy indicates 100% spin polarization in the Mn-doped Ge-core/Si-shell nanowire.

**Fig. 5:**
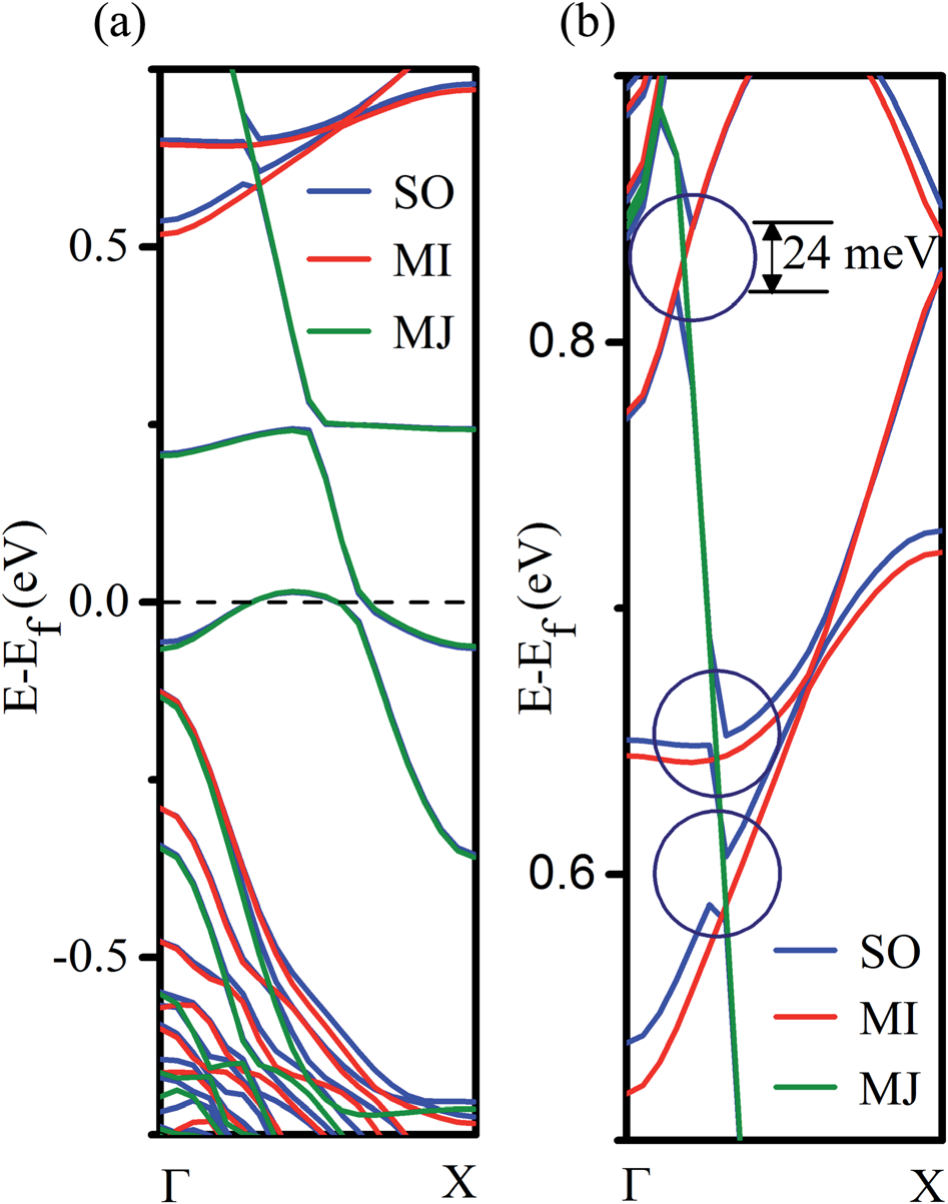
(a) The electronic band structure (PBE) of the Mn-doped Ge-core/Si-shell nanowire with and without spin–orbit (SO) coupling; MJ and MI refer to the majority and minority spin directions. (b) Magnified version of (a) depicting SO splitting at the crossing points of MJ and MI bands.

To further investigate the effect of spin–orbit interaction on the energy bands of the Mn-doped (site I) nanowire, we have performed the spin-unconstrained noncollinear DFT calculations that include the spin–orbit coupling (SOC) effect. Our results reveal that the inclusion of the SOC does not alter the half-metallic properties of our system, but as expected, it lifts the degeneracies at the crossings points of energy bands for the majority and minority spin carriers as shown in Fig. 5a. The maximum SO splitting at the band crossing is found to be ∼24 meV (Fig. 5b). However, no measurable shifts in energy levels in the vicinity of Fermi energy are observed due to spin–orbit coupling, which suggests that the spin coherence length can be much higher in this material.

Thus far, we have focused only on the energetically most stable structure of the Mn-doped nanowire (Mn at site I) and its properties. However, the doping or implantation of Mn into the Ge-core\Si-shell nanowire is a non-equilibrium process. Hence, Mn may occupy other possible sites in the core as well as in the core and shell. To examine these possibilities, we have calculated the electronic band structure for Mn dopants at various substitutional sites (see Fig. S4–S6 in the ESI†). A half-metallic feature is clearly noticeable in all these cases. Increasing the concentration of Mn from 0.78% to 1.56% does not alter the half-metallic properties (see Fig. S6 and S7 in the ESI†). We have also studied the case in which Mn is at the interstitial site. Though the half-metallic behavior is still observed, the minority spin electrons exhibit a semiconducting behavior with an indirect bandgap of 0.07 eV (see Fig. S8 in the ESI†).

To investigate the effect of the external strain, which may arise during synthesis of nanowires at finite temperature, we have also calculated the electronic band structure of the Mn-doped nanowire (Mn at site I) for both the tensile and compressive strain. To model the tensile or compressive strain, we have varied the lattice parameter (*a*) appropriately from its equilibrium value (7.92 Å) and allowed the atomic structure to relax without symmetry constraint until the residual force on each atom becomes less than 0.01 eV Å^−1^. As seen from the electronic band structure (see Fig. S9 in the ESI†), the system is half-metallic in nature under tensile strain values of +1.26% and +2.52%. The minority spin electrons display a semiconducting behavior in both the cases with direct energy gaps of 0.74 eV and 0.81 eV respectively. The majority spin carriers, on the other hand, show a metallic characteristic. Our calculations reveal that the tensile strain along the nanowire axis is found to increase the bandgap in the minority spin direction. In the case of compressive strain (see Fig. S10 in the ESI†), a half-metallic feature is clearly noticeable at a strain of −1.26%. The minority spin electrons exhibit a semiconducting behavior with a direct energy gap of 0.55 eV, whereas the majority spin carriers show a metallic behavior. However, for a large compressive strain of −2.52%, a semiconductor to metal phase transition in the minority spin direction is observed.

**Fig. 6:**
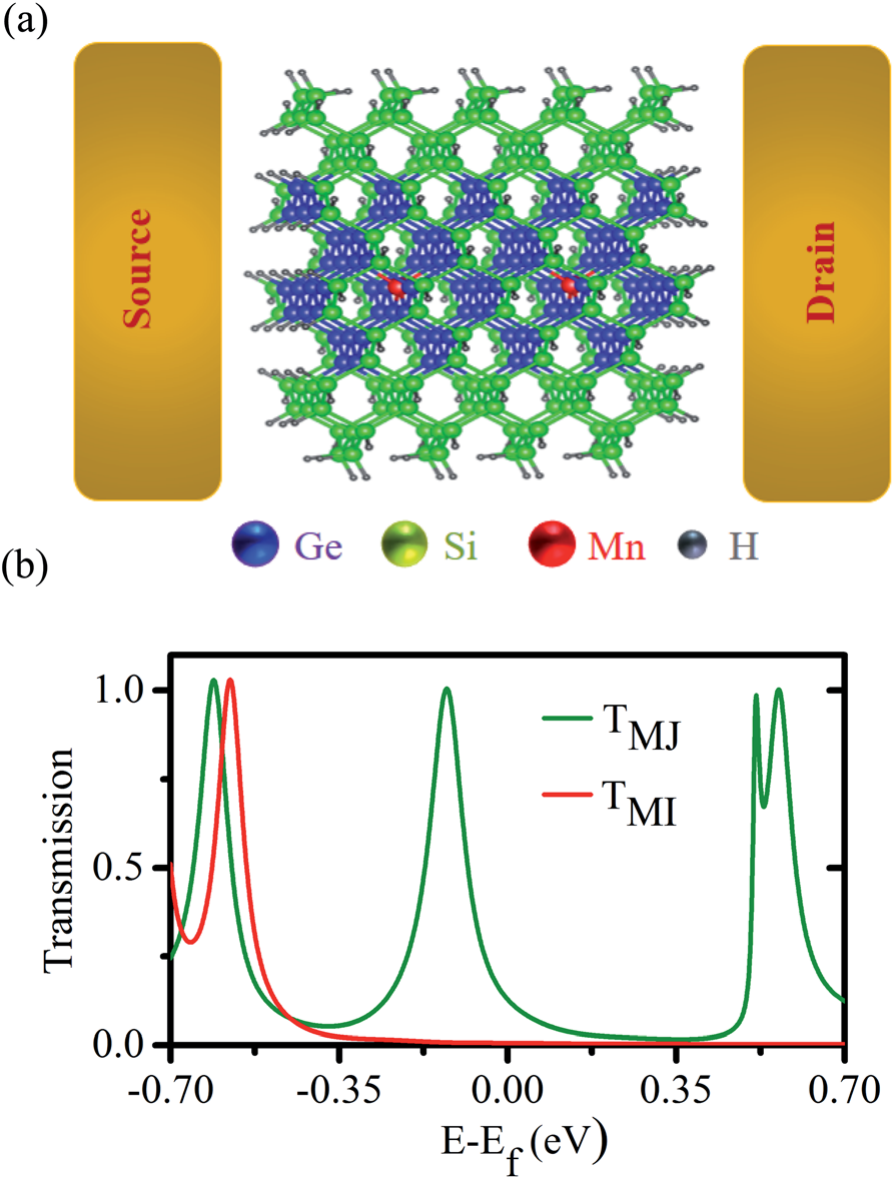
(a) A prototypical Mn-doped Ge-core/Si-shell nanowire spin-filter; the channel length is ∼2.37 nm (electrode–electrode distance). (b) Calculated spin-dependent transmission; *T*_MJ_ and *T*_MI_ refer to the transmission for the majority and minority spin carriers, respectively.

Next, to access the spin-filtering properties of the Mn-doped Ge-core/Si-shell nanowire, we have constructed a prototypical Mn-doped Ge/core–Si-shell nanowire junction (Mn at site I) as shown in Fig. 6a. A spin conserved tunneling approach^41^ is used to calculate the transmission function for the majority (*T*_MJ_) and minority (*T*_MI_) spin carriers (Fig. 6b). Due to confinement of carriers to the spin active Ge-core and observed weak spin–orbit interaction in the Mn-doped Ge-core/Si-shell nanowire, we have assumed the scattering to be coherent and neglected the spin-flip scattering effect in our calculations. Fig. 6b shows that there are no transmission peaks found for the minority spin carriers in the vicinity of Fermi energy. However, a transmission peak appears close to Fermi energy for the majority spin carriers. Analysis of orbital coefficients reveals that the p states of Ge and the d, as well as p, states of Mn that couple to the s and p states of the gold electrode contribute to the transmission peak in the spin majority case. In the case of minority spins, the metal-induced broadening is responsible for an insignificant but a finite transmission value of 6.5 × 10^−3^ at the Fermi energy. The *T*_MJ_ at the Fermi energy is found to be 128.9 × 10^−3^. To quantify the asymmetry in spin-dependent transmission, we have calculated the spin-filter efficiency,^55^
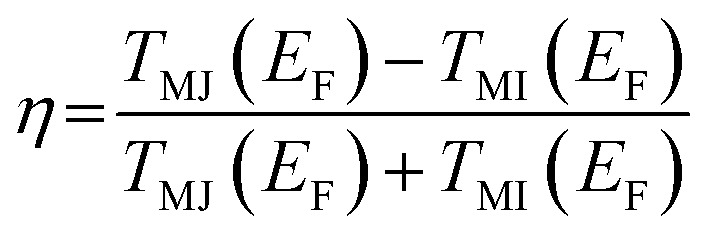
 using the transmission values for the majority and minority spin carriers at the Fermi energy. The value for *η* is found to be 90.4%, which unambiguously confirms the spin-selective properties of the Mn-doped Ge-core/Si-shell nanowire channel. We expect the *η*-value to reach 100% with an increase in the channel length of the nanowire as the transmission of the minority carrier with a semiconducting feature would fall exponentially^56^ with an increase in the length of the channel. An increase of spin-filtering efficiency has been reported with the increase of channel length in other materials.^55^.

## Conclusions

In summary, we predict that a small amount of Mn dopants in the core region of a Ge-core/Si-shell nanowire can transform the semiconducting Ge–Si core–shell nanowire into a half-metallic ferromagnet with 100% spin polarization at the Fermi energy. The ferromagnetic spin ordering is found to be stable at room temperature. The spin-unconstrained non-collinear magnetic calculation that includes spin–orbit interaction reveals no measurable shift in energy levels in the vicinity of the Fermi energy, which suggests that the spin-coherence length can be much larger in this material. The high spin filter efficiency (>90%) obtained using a quantum transport approach in a prototypical nanowire junction further confirms the spin-selective properties of this material. We expect that this new finding will attract experimental interest towards this material for possible application in room temperature spintronics.

## Conflicts of interest

The authors declare no competing financial interest.

## Supplementary Material

NA-002-C9NA00803A-s001
