# Supplementary material for: Spin filtering with Mn-doped Ge-core/Si-shell nanowires
Source: Nanoscale Adv. 2020 Feb 28;2(5):1843–9. doi: 10.1039/c9na00803a (PMC9416944; doi:10.1039/c9na00803a)
Supplement: NA-002-C9NA00803A-s001 [file NA-002-C9NA00803A-s001.pdf]

Electronic Supplementary Material (ESI) for Nanoscale Advances.
This journal is © The Royal Society of Chemistry 2020

Supporting Information

Spin Filtering with Mn-doped Ge-core/Si-shell Nanowire

Sandip Aryal and Ranjit Pati

Department of Physics, Michigan Technological University, Houghton, MI 49931, USA

*E-mail: patir@mtu.edu

Contents

Figure S1. The top view of the relaxed Mn-doped Ge-core/Si-shell nanowire along the <110> direction with Mn-doped (**site I**) in the core.

Figure S2. Atom decomposed electronic band structure (PBE) of relaxed Mn-doped (**site I**) Ge-core/Si-shell nanowire.

Figure S3. Atom decomposed electronic band structure (HSE06) of relaxed Mn-doped (**site I**) Ge-core/Si-shell nanowire.

Figure S4. The optimized structure and electronic energy band diagram (PBE) of Mn-doped (**site II**) Ge-core/Si-shell nanowire.

Figure S5. The optimized structure and electronic energy band diagram (PBE) of Mn-doped (**site III**) Ge-core/Si-shell nanowire.

Figure S6. The optimized structure and electronic energy band diagram (PBE) of Mn-doped (**both core and shell**) Ge-core/Si-shell nanowire.

Figure S7. The optimized structure and electronic energy band diagram (PBE) of Mn-doped (**two Mn atoms per unit cell at site I**) Ge-core/Si-shell nanowire.

Figure S8. The optimized structure and electronic energy band diagram (PBE) of Mn-doped (**interstitial site**) Ge-core/Si-shell nanowire.

Figure S9. Electronic band structure (PBE) of Mn-doped (**site I**) Ge-core/Si-shell nanowire under the tensile strain along the nanowire axis.

Figure S10. Electronic band structure (PBE) of Mn-doped (**site I**) Ge-core/Si-shell nanowire under the compressive strain along the nanowire axis.

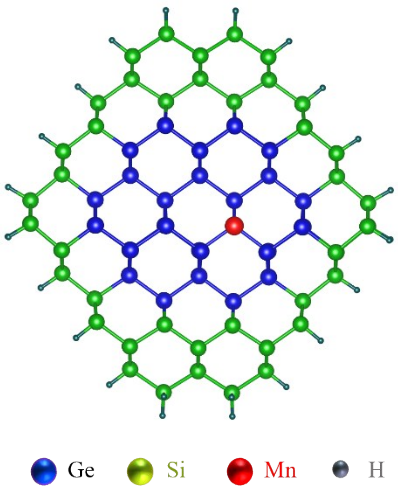

Figure S1. The top view of the optimized Mn-doped (site I) Ge-core/Si-shell nanowire along the <110> direction. The core diameter of the nanowire is 11.7 Å; the unsaturated surface states are passivated by H-atoms.

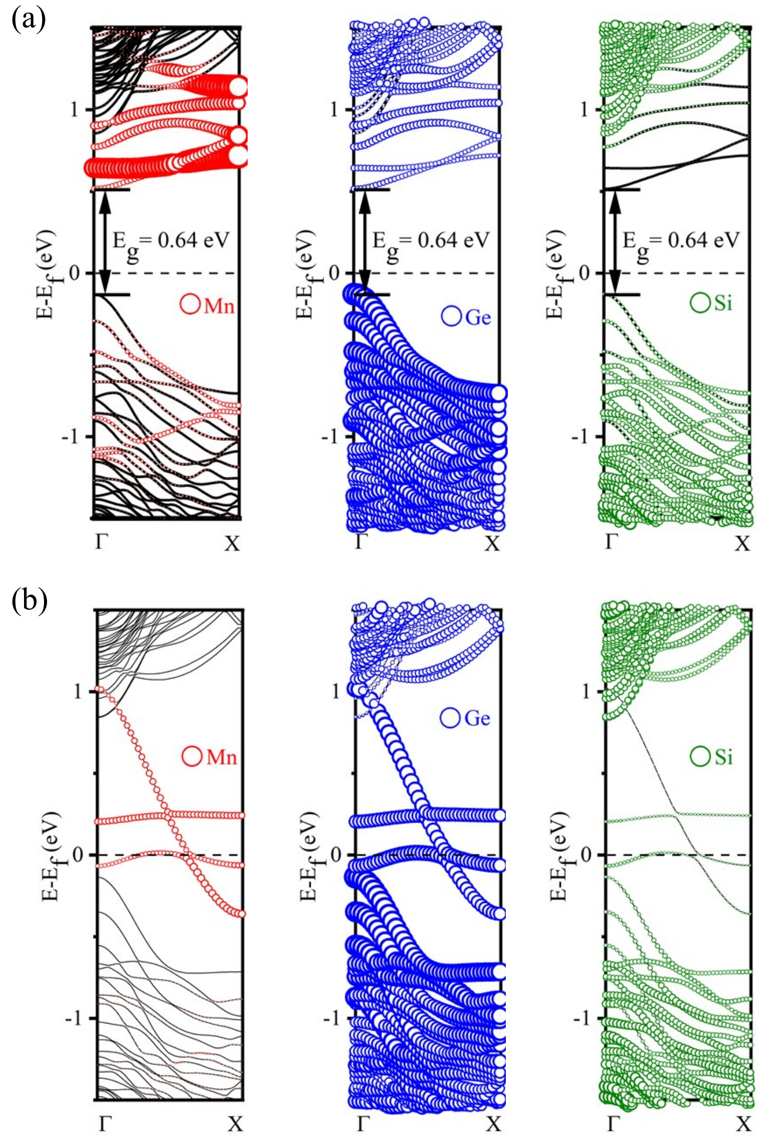

Figure S2. Atom decomposed electronic band structure (PBE) of optimized Mn-doped Ge-core/Si-shell nanowire (Mn at site I) showing the contributions of the individual atoms (Mn, Ge, Si): (a) Minority-spin direction, (b) Majority-spin direction. The minority spin electrons exhibit semiconducting feature with a direct energy gap of 0.64 eV. The Ge atoms contribute to the valence band in the minority spin direction at the г point. Both Mn and Ge contribute to the conduction band at the г point. The majority spin electrons, however, show metallic characteristic with contributions from both Mn and Ge atoms at the Fermi energy; larger circle size implies larger contribution.

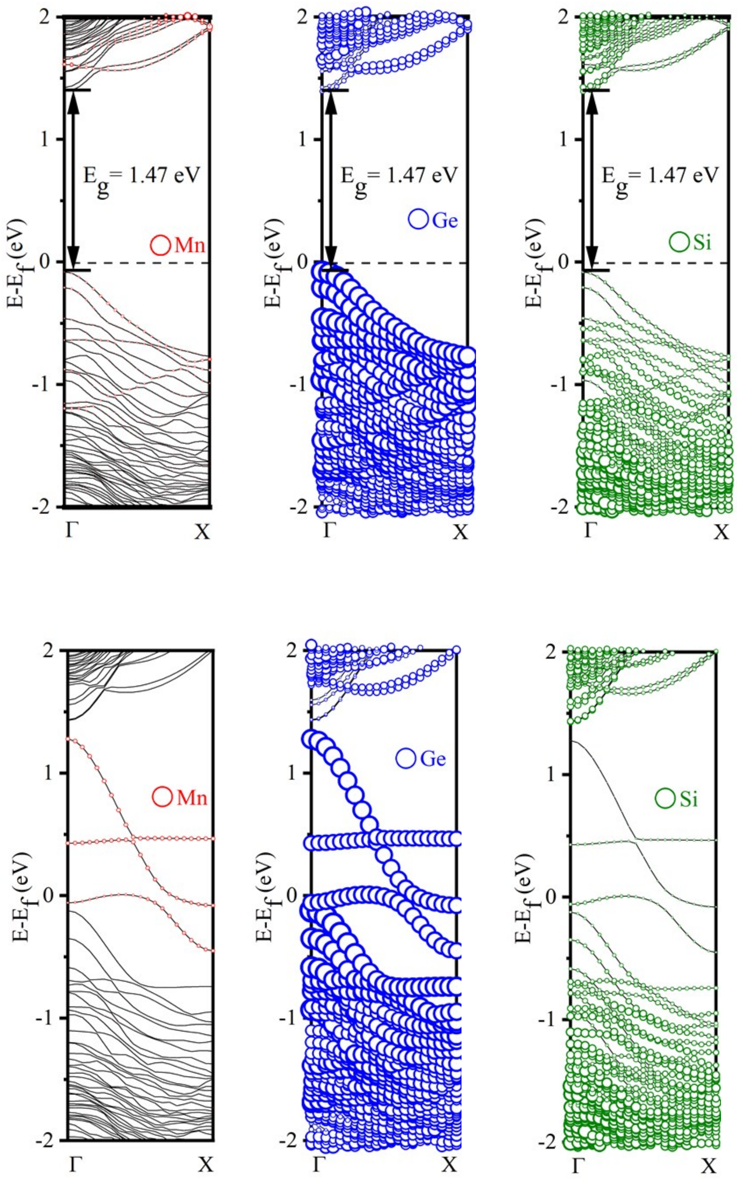

Figure S3. Atom decomposed electronic band structure (HSE06) of optimized Mn-doped Ge-core/Si-shell nanowire (Mn at site I) showing the contributions of the individual atoms (Mn, Ge, Si): (a) Minority-spin direction, (b) Majority-spin direction. The minority spin electrons exhibit semiconducting feature with a direct energy gap of 1.47 eV. The Ge atoms contribute to the valence band in the minority spin direction at the Γ point. Both Si and Ge contribute to the conduction band at the Γ point. The majority spin electrons, however, show metallic characteristic with contributions from both Mn and Ge atoms at the Fermi energy; larger circle size implies larger contribution.

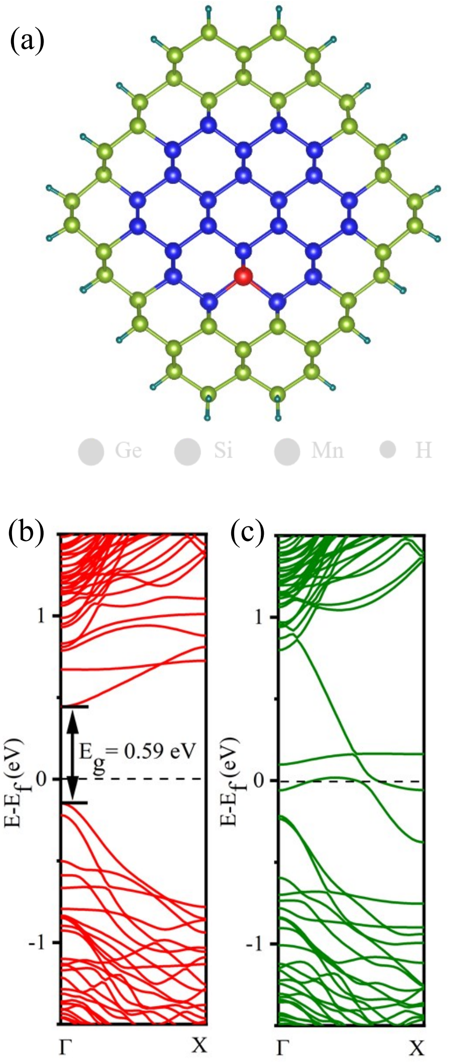

Figure S4. (a) The top view of the optimized Mn-doped Ge-core/Si-shell nanowire along the <110> direction (Mn at the site II). Electronic band structure (PBE) of Mn-doped Ge-core/Si-shell nanowire: (b) Minority-spin direction, (c) Majority-spin direction. The half-metallic feature is clearly noticeable.

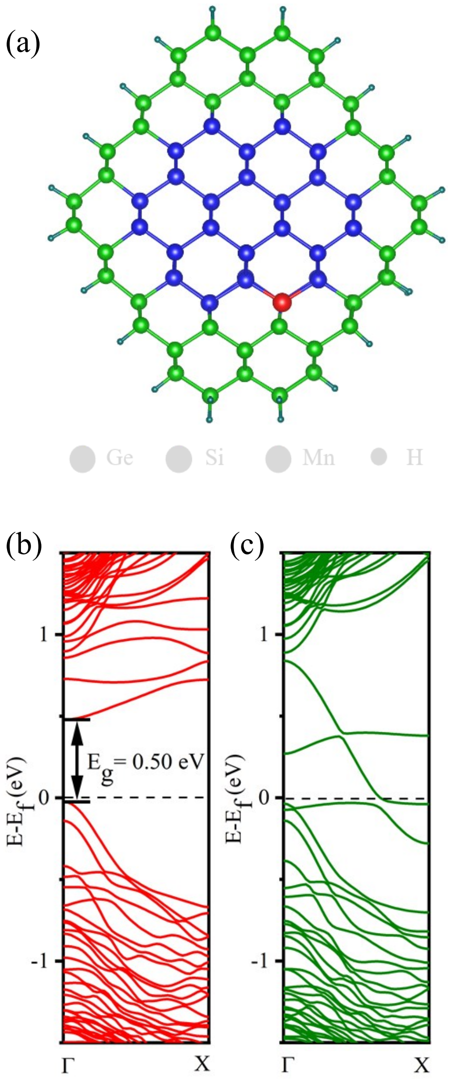

Figure S5. (a) The top view of the optimized Mn-doped Ge-core/Si-shell nanowire along the <110> direction (Mn at the site III). Electronic band structure (PBE) of Mn-doped Ge-core/Si-shell nanowire: (b) Minority-spin direction, (c) Majority-spin direction. The half-metallic feature is clearly noticeable.

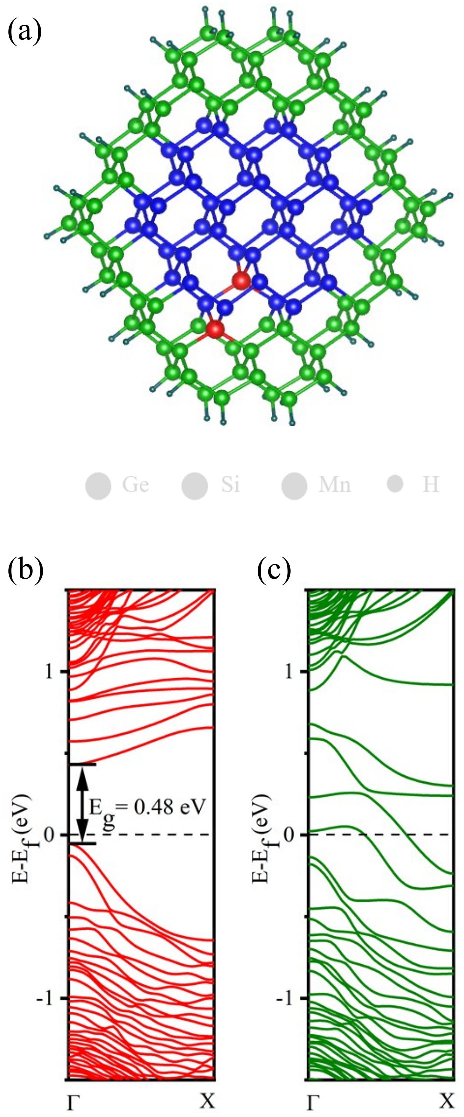

Figure S6. (a) The top view of the optimized Mn-doped Ge-core/Si-shell nanowire along the <110> direction with a Mn atom doped (substitutional) in both core and shell. Electronic band structure (PBE) of Mn-doped Ge-core/Si-shell nanowire: (b) Minority-spin direction, (c) Majority-spin direction. A half-metallic feature is clearly noticeable. The minority spin carriers exhibit semiconducting characteristics with a direct energy gap of 0.48eV. The majority spin carriers, on the other hand, show metallic behavior.

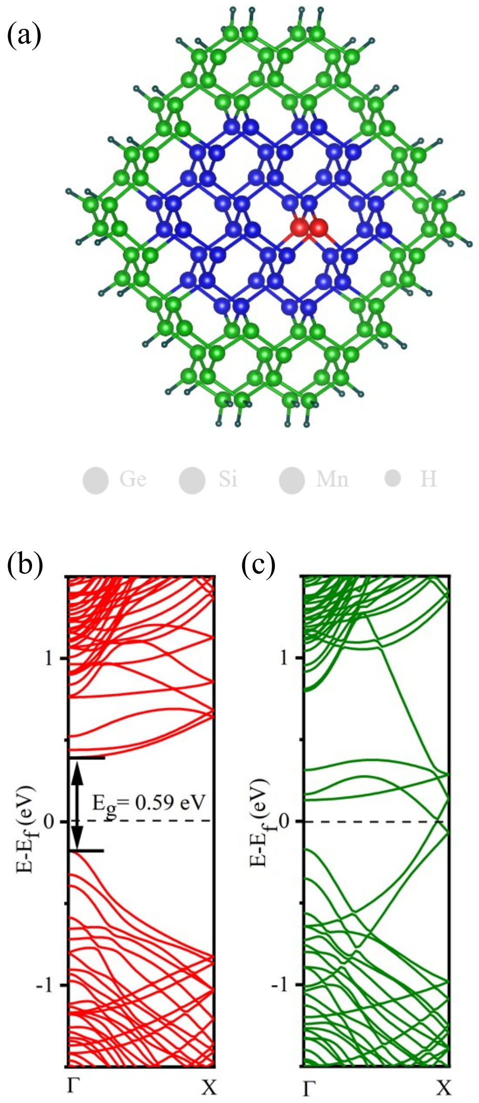

Figure S7. (a) The top view of the optimized Mn-doped Ge-core/Si-shell nanowire along the <110> direction with two Mn atoms (substitutional site I) per unit cell in the core. Electronic band structure (PBE) of the Mn-doped Ge-core/Si-shell nanowire: (b) Minority-spin direction, (c) Majority-spin direction. A half-metallic feature is clearly noticeable. Increase in Mn concentration from 0.78% to 1.56% is found to decrease the energy bandgap by 0.05 eV in the minority spin direction.

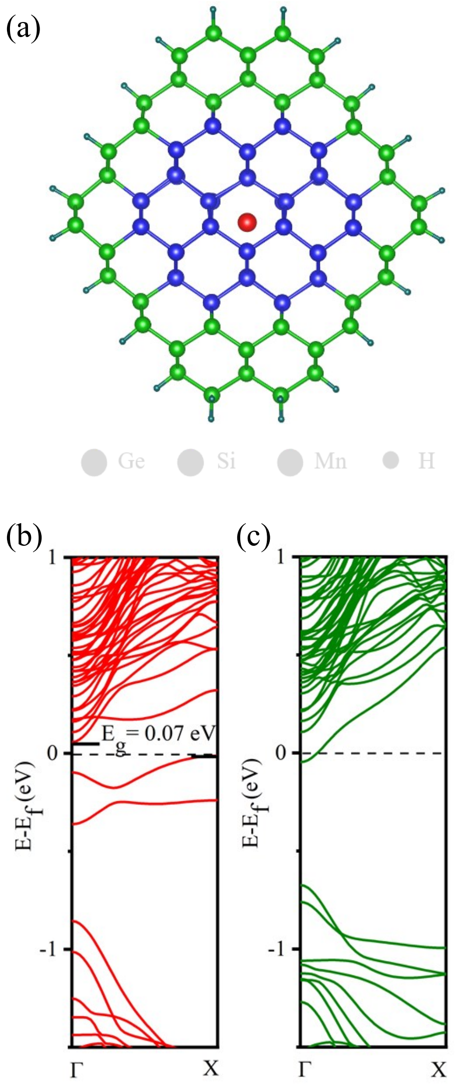

Figure S8. (a) The top view of the optimized Mn-doped Ge-core/Si-shell nanowire along the <110> direction (Mn at the interstitial site). Electronic band structure (PBE) of Mn-doped Ge-core/Si-shell nanowire: (b) Minority-spin direction, (c) Majority-spin direction. The half-metallic feature is noticeable. The minority spin electrons exhibit a semiconducting behavior with an indirect energy gap of 0.07 eV, whereas the majority spin carriers show a metallic behavior. Compared to site I (substitutional), there is a significant decrease in bandgap for the minority spin direction in the case of interstitial doping.

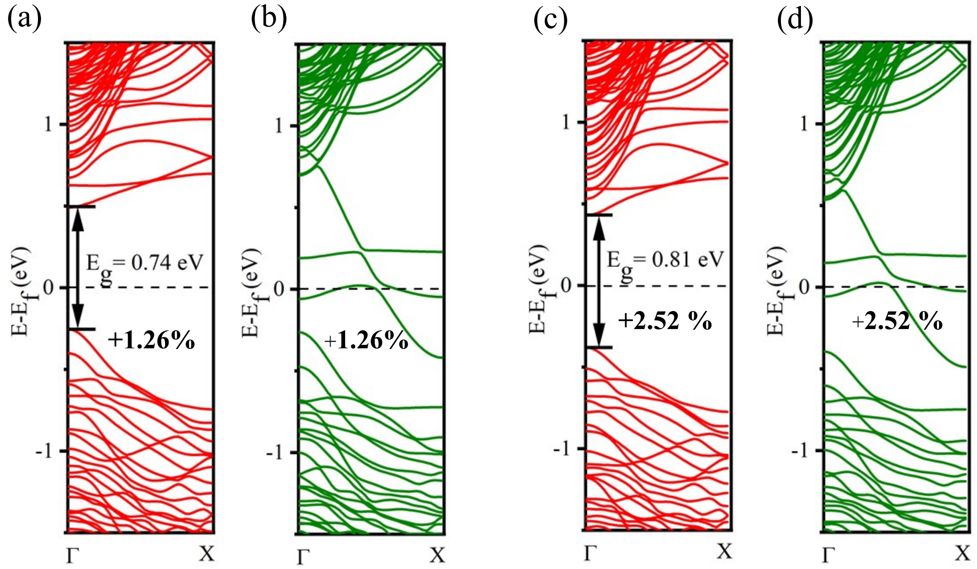

Figure S9. Electronic band structure (PBE) of Mn-doped Ge-core/Si-shell nanowire (Mn at the site I) under tensile strain along the nanowire axis. (a) and (b) represent the band structure in the Minority and Majority-spin direction respectively under lateral strain of +1.26%. (c) and (d) represent the band structures in the Minority and Majority-spin direction respectively under lateral strain of +2.56%.

As seen from the electronic band structure (Figure S9), the system is half-metallic in nature under tensile strain values of +1.26% and +2.52%. The minority spin electrons (Figure S9 (a) and (c)) display a semiconducting behavior in both the cases with a direct energy gap of 0.74 eV and 0.81 eV respectively. The majority spin carriers (Figure S9 (b) and (d)), on the other hand, show a metallic characteristic. Our calculations reveal that the tensile strain along the nanowire axis is found to increase the band gap in the minority spin direction.

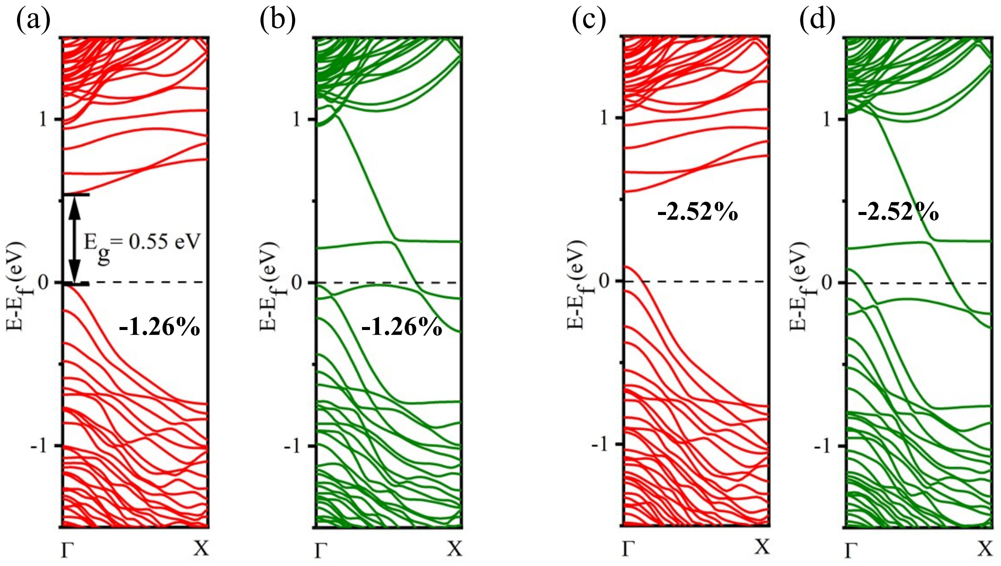

Figure S10. Electronic band structure (PBE) of Mn-doped Ge-core/Si-shell nanowire (Mn at site I) under compressive strain along the nanowire axis. (a) and (b) represent the band structure in the Minority and Majority-spin direction respectively under compressive strain of -1.26%; (c) and (d) represent the band structures in the Minority and Majority-spin direction respectively under compressive strain of -2.52%.

A half-metallic feature is clearly noticeable at the compressive strain of -1.26%. The minority spin electrons (Figure S10 (a)) exhibit a semiconducting behavior with a direct energy gap of 0.55 eV. The majority spin carriers (Figure S10 (b)), on the other hand, show a metallic behavior. For compressive strain of -2.52%, there is a semiconductor to metal phase transition in the minority spin direction (Figure S10 (c)).
